# Strategies and evaluation underpinning the implementation of suicide prevention training: a systematic review

**DOI:** 10.1186/s12889-025-21999-8

**Published:** 2025-03-06

**Authors:** Adelia Khrisna Putri, Martina McGrath, Rachel Batchelor, Victoria Ross, Karolina Krysinska, Jacinta Hawgood, Kairi Kõlves, Lennart Reifels, Jane Pirkis, Karl Andriessen

**Affiliations:** 1 Centre for Mental Health and Community Wellbeing, Melbourne School of Population and Global Health, The University of Melbourne, Melbourne, VIC Australia; 2 Faculty of Psychology, Universitas Gadjah Mada, Yogyakarta, Indonesia; 3 The Oxford Institute of Clinical Psychology Training and Research, University of Oxford, Oxford, UK; 4 Australian Institute for Suicide Research and Prevention, School of Applied Psychology, Griffith University, Brisbane, QLD Australia

**Keywords:** Suicide prevention training, Implementation, Evaluation, Systematic review

## Abstract

**Background:**

Suicide prevention training programs can enhance the capacity for suicide prevention by improving the attitudes and comprehension of individuals regarding suicide and increasing their skills in supporting a suicidal person. However, little is known about how training programs are implemented and how implementation is assessed. Thus, our review aims to identify the strategies and evaluation methods underpinning the implementation of suicide prevention training programs.

**Methods:**

The systematic review adhered to the PRISMA guidelines and involved searches in MEDLINE, Embase, Emcare, PsycINFO, EBM Reviews, Scopus, and a forward and backward citation search following the full-text screening. Eligible studies (*n* = 28) reported the implementation strategy or implementation evaluation of a suicide prevention training program (PROSPERO #CRD42021288621).

**Results:**

The implementation strategies varied among three categories of training programs. Gatekeeper training predominantly utilized a train-the-trainer format and collaborations with stakeholders. Professional development training focused more on establishing supportive organizational infrastructure and extended post-training supervision. School-based curriculum training programs emphasized the distribution of educational materials and role-play activities. Surveys were the primary evaluation method, often complemented by interviews, observations, progress tracking, or focus groups. Evaluations primarily assessed acceptability, fidelity, and feasibility.

**Conclusion:**

While certain training categories tend to employ specific strategies and evaluation measures more frequently than others, stakeholder collaboration, assessing content relevance, and follow-up supervision could be valuable across training programs. Tailored strategies may cater for groups with varying levels of knowledge and training in suicide prevention to enhance acceptability and feasibility. Future research should evaluate approaches that facilitate adoption and sustainability of suicide prevention training programs.

**Supplementary Information:**

The online version contains supplementary material available at 10.1186/s12889-025-21999-8.

## Introduction

Suicide is a significant global public health concern [1], and effective implementation of suicide prevention strategies is vital for reducing suicide mortality [2]. Suicide prevention training programs are a critical component of these strategies, equipping individuals with the knowledge and skills to identify and intervene in situations involving suicide risk [3]. These programs vary widely in design, implementation strategies, and target populations, allowing them to be adapted to specific needs.

Examples include Question, Persuade, and Refer (QPR) [4, 5] and Applied Suicide Intervention Skills Training (ASIST) [6], which are widely implemented in various settings. The QPR Institute provides training tailored for individuals, organizations, and professionals (https://qprinstitute.com/), while ASIST offers intensive workshops tailored for diverse groups, such as children, youth, and Indigenous communities (https://www.suicideinfo.ca/workshops/). Together, these programs fall under the broad category of suicide prevention training, which can be further categorized by target populations and training goals.

One of the most common types of suicide prevention training programs is gatekeeper training, which targets individuals with direct contact with those at risk of suicide. Gatekeepers are traditionally divided into two groups: trained professionals (e.g., psychologists, doctors, social workers) and community members without formal intervention training (e.g., teachers, police, counselors) who can play critical roles [7]. Over time, however, it is more often used to describe training for community members. Programs like QPR are commonly designed to improve community members’ ability to recognize warning signs, engage with at-risk individuals, and provide referrals [8].

Meanwhile, professional development training is tailored for clinical and non-clinical professionals, offering intensive or refresher training through supervision, workshops, or seminars. Research has shown that general practitioners (GPs) often feel unconfident managing depression and suicide in daily practice [9], despite frequently being the first point of contact for individuals who die by suicide in the weeks before their death. Programs like the E-Collaborative Assessment and Management of Suicidality (e-CAMS) have been proven to enhance health professionals' skills in assessing, monitoring, and intervening with suicidal patients [10]. Similarly, the OSPI-Europe training program, delivered to 208 GPs across three European countries, improved participants' attitudes toward suicide prevention and their confidence in handling suicide cases [9]. However, the study noted that only the increased confidence was sustained at follow-up.

Another type of suicide prevention training is school-based programs, where interventions are integrated into curricula to educate students and staff on identifying and addressing suicide risk [11]. One effective example is the Youth Aware of Mental Health (YAM) program, a universal school-based intervention shown to significantly improve help-seeking behaviors and mental health literacy [12, 13], while reducing suicidal ideation [13]. However, some limitations have been noted. For example, a study with 436 adolescents reported no significant change in their intent to seek help after the training [12]. Additionally, while YAM improved help-seeking behaviors with peers and school staff, it did not result in significant changes in help-seeking behavior with mental health professionals over time.

These training types highlight the diverse approaches to suicide prevention across various populations and contexts. However, each type has its limitations despite its positive effects. For example, a systematic review of 28 studies on gatekeeper training, such as QPR for community members, found a decline in knowledge over time. Additionally, in 57% of studies evaluating gatekeeper attitudes (4 out of 7), attitudes returned to baseline levels by follow-up [14]. These findings suggest that while gatekeeper programs can effectively enhance short-term skills and attitudes, their long-term sustainability may require ongoing support, such as booster sessions or refresher training.

In contrast, programs like ASIST, delivered to 543 helping professionals in Lithuania, have demonstrated better long-term skill retention, particularly when paired with follow-up training [15]. These observations highlight the importance of evaluating suicide prevention training programs beyond their immediate effects. Understanding how programs are implemented (e.g., strategies used) and how their implementation is evaluated (e.g., outcomes of implementation) is essential to improving their long-term effectiveness.

Implementation strategies refer to “methods or techniques used to enhance the adoption, implementation, and sustainability of a clinical program or practice” [16]. These strategies include, among others, train-the-trainer approaches, identifying champions (individuals in community who actively promote the program) and early adopters (first individuals or groups to try out and implement a new program), and working with educational institutions [16]. Implementation outcomes are effects of deliberate and purposive actions to implement a training program, such as acceptability (the extent to which the training program is perceived as satisfactory, appropriate, or agreeable by its stakeholders), fidelity (the degree to which the training program is delivered as intended by its developers), and adoption (initial program uptake) [17]. One of the benefits of evaluating implementation strategies is that it allows us to explore how a training program could be further improved (e.g., regarding specific components, delivery mechanisms, or duration) to meet the needs of a particular target group [18]. The aim of this review is to identify the strategies and evaluation methods underpinning the implementation of suicide prevention training programs.

### Objectives

This systematic review set out to achieve the following objectives:

1. Identify the types of implementation strategies used in suicide prevention training programs, including their target population, training type, aim, dose, actor, and delivery strategies.
2. Explore the evaluation methods employed to assess the implementation of suicide prevention training programs, including specific measures and tools.
3. Examine the outcomes measured in these programs.
4. Identify factors that contribute to the effectiveness of the suicide prevention training implementation, including barriers and facilitators.

## Methods

**Fig. 1:**
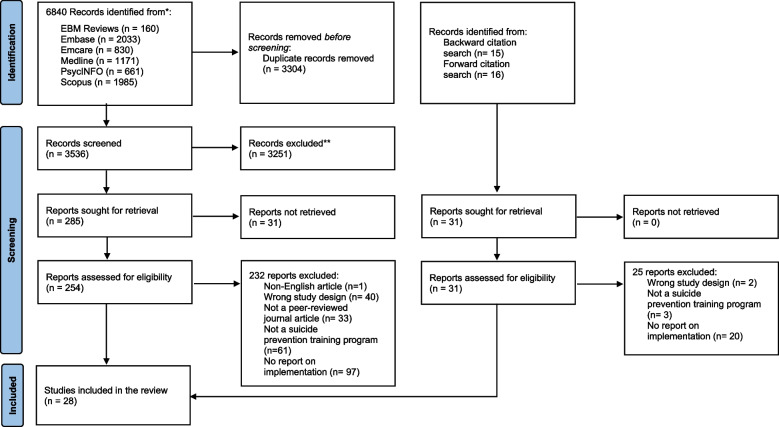
PRISMA flow diagram. Adapted from Page MJ, McKenzie JE, Bossuyt PM, Boutron I, Hoffmann TC, Mulrow CD, et al. The PRISMA 2020 statement: an updated guideline for reporting systematic reviews [19]

This review adhered to the PRISMA guidelines [19] and the protocol was registered in PROSPERO (CRD42021288621). We built a search string comprising the concepts of suicide prevention, training, and implementation and conducted searches in the following databases: MEDLINE, Embase, Emcare, PsycINFO, EBM Reviews, and Scopus. Medline was searched using MeSH and search words: (suicide.mp OR Suicide/ OR suicide prevention.mp. OR suicide intervention.mp. OR suicide assessment.mp. OR suicide screening.mp. OR suicide management.mp. OR postvention.mp. OR Bereavement/ or bereave*.mp. OR Grief/ or grief.mp. OR Suicide loss.mp.) AND (training.mp. OR gatekeeper training.mp. OR professional training.mp. OR education.mp. OR Education/ OR curriculum.mp. OR Curriculum/) AND (Implementation Science/ OR implementation.mp. OR adherence.mp. OR delivery.mp. OR disseminat*.mp. OR fidelity.mp. OR mechanisms.mp. OR process evaluation.mp. OR responsive.mp. OR standard* delivery.mp. OR train the trainer.mp. OR transfer.mp. OR translation.mp.). We used the same search string in the other databases using subject headings and keywords. The searches were limited to studies published in English in peer-reviewed journals, but not by location or date of publication. We checked the reference lists of the selected studies and conducted forward citation searches of the selected studies to identify additional relevant studies through Google Scholar. Figure 1 presents the search and selection process.

KA conducted the searches in April 2022, exported the results into EndNote, and removed duplicates. VR and KK independently conducted a pilot screening of 10% of the references based on title and abstract. The interrater reliability was substantial (Cohen’s k = 0.60). Then, VR and KK each screened half of the references based on title and abstract. Next, RB and MM independently conducted a full text screening of the selected abstracts against the inclusion and exclusion criteria. There was moderate agreement between the screeners (Cohen’s k = 0.59), and disagreements were resolved by KA. AKP updated the searches in February 2023. AKP and MM independently conducted the title and abstract, and full text screening of the updated search, and KA resolved any disagreement between the two screeners.

### Inclusion and exclusion criteria

Studies were included if they: (i) reported on a suicide prevention training program, (ii) used qualitative, quantitative, or mixed methods, (iii) provided empirical data regarding the implementation or implementation evaluation of a suicide prevention training program, and (iv) and were published in English in a peer-reviewed journal.

Studies were excluded if they: (i) did not report on a suicide prevention training program, (ii) used other methods such as case studies or literature review, and (iii) did not provide data regarding the implementation or evaluation of a suicide prevention training program.

### Data extraction

AKP and MM independently extracted the following data from the selected studies: authors, year, location of study, eligibility criteria, sample size, participants’ age and gender distribution, type of training, aim of training, training setting, duration of the training, implementation strategy, methods of evaluation, measures used in the evaluation, and main outcomes of the implementation. KA resolved disagreements between the two screeners.

### Quality assessment

We assessed the methodological quality of the included studies using the Mixed Methods Appraisal Tool (MMAT) [20]. This instrument is a flexible tool used to evaluate the methodological quality of five types of study designs: qualitative, randomized controlled trials, non-randomized studies, quantitative descriptive studies, and mixed-method studies. The MMAT does not have a cut-off score to characterize low- and high-quality studies. Instead, its purpose is to highlight which part of the study could still be lacking so readers can take it into consideration when interpretating the result. MM and AKP independently conducted the quality assessment. The assessment results were then compared, and KA resolved any disagreements.

## Results

### Study characteristics

The database searches yielded 28 eligible papers published between 1999 and 2023. There were three pairs of studies that were reported in two papers each: one implemented ASIST training [6, 21], one on PC CARES training [22, 23], and the third one on interprofessional education (IPE)-based suicide prevention [24, 25]. In this review we report on the individual papers unless otherwise stated. Nineteen papers reported studies conducted in the USA [5, 6, 8, 10, 11, 21–34], four in Australia [35–38], and one in each of the following countries: Canada [39], Scotland [40], the United Kingdom [41], and Guyana [4]. One paper reported on data collected from multiple countries, namely Germany, Hungary, Portugal, and Ireland [42]. Eighteen suicide prevention training programs were reported across these papers (Table 1), with QPR training being the most reported training program (*n* = 5, 28%).

**Table 1:** List of suicide prevention training programs

No	Prevention Training Program	Number of Studies
1	QPR^a^	5
2	ASIST^b^	2
3	IPE-informed suicide prevention course^c^	2
4	PC CARES^d^	2
5	Regional suicide prevention training program^e^	2
6	SPI^f^	2
7	STORM^g^	2
8	YAM^h^	2
9	ASAP^I^	1
10	CAMS-G^J^	1
11	e-CAMS^K^	1
12	Guideline implementation training^L^	1
13	OSPI-Europe 4-level suicide prevention program^M^	1
14	SOS^N^	1
15	Sources of Strength^O^	1
16	Suicide story training^P^	1
17	We Yarn Training^Q^	1
18	VitalCog Training^R^	1

^a^QPR: Question, Persuade, Refer [4, 5, 8, 34, 39]

^b^ASIST: Applied Suicide Intervention Skills Training [6, 21]

^c^IPE: Interprofessional Education [24, 25]

^d^PC-CARES: Promoting Community Conversations About Research to End Suicide [22, 23]

^e^The study did not specify the name of the training

^f^SPI: Safety Planning Intervention [27, 31]

^G^STORM: Skills Training on Risk Management [40, 41]

^h^YAM: Youth Aware of Mental Health [11, 26]

^I^ASAP: Adolescent Suicide Awareness Program [29]

^J^CAMS-G: Collaborative Assessment and Management of Suicidality – Group [28]

^K^e-CAMS: E-Collaborative Assessment and Management of Suicidality [10]

^L^Unspecified regional suicide prevention training [36, 37]

^M^Multi-region suicide prevention training [42]

^N^SOS Signs of Suicide [32]

^O^Peer-led suicide prevention intervention [30]

^P^Training with narrative therapy approach [38]

^Q^Culturally safe suicide prevention training [35]

^R^Virtual suicide prevention training [33]

Table 2 summarizes the characteristics of the included papers. The papers reported on studies that were mainly conducted within the education setting, including seven papers within a secondary school setting (24%) and three at the university level (*n* = 3; 10%). Ten papers reported on studies conducted within health and mental healthcare settings, including mental health facilities (*n* = 3; 10%), hospitals (*n* = 1; 3%), crisis centers (*n* = 2; 7%), remote health centers (*n* = 1; 3%) and veteran affairs centers (*n* = 3, 10%). There were also papers about a more specific setting, such as prisons (*n* = 1; 3%), workplaces (*n* = 1, 3%) and youth-serving agencies (*n* = 1; 3%). The remaining papers were on studies in a broader context within the community (*n* = 4; 10%) and at the regional level (*n* = 2; 7%).

**Table 2:** Study characteristics

No	Authors	Eligibility Criteria	Sample Size	Age (years), M, SD, Range	Gender	Setting	Type of Training
1	Bailey et al. (2021) USA [26]	Students attending rural schools	*N* = 269 (baseline) *N* = 217 (baseline and follow-up)	Avg 15.7 ± .75 years	Female (52.1%) LGBTQ (5.5%)	Rural schools	Curriculum Training
2	Bettis et al. (2020) USA [27]	Psychiatry and psychology trainees, nursing and social work staff	*N* = 30	N/A	N/A	Paediatric behavioural health emergency services	Professional Development
3	Cerel et al. (2012) USA [8]	Kentucky community members who received QPR (Question Persuade Refer) training	*N* = 3,958	Age range: 18 – 84 (M = 36.72; SD = 13.52)	Female (*n* = 2,822, 71%) Male (*n* = 1,136, 29%)	Community	Gatekeeper
4	Cramer et al. (2019) USA [25]	Health profession students	*N* = 19	Young adult age range (M = 23.16; SD = 2.67)	Female (*n* = 17, 89.5%)	Universities	Professional Development
5	Cross et al. (2014) USA [6]	Counsellors who had received training for delivering ASIST at 17 Lifeline crisis centers	*N* = 34 (trainers)	N/A	N/A	Crisis centres	Gatekeeper
6	Cross et al. (2017) USA [21]						
7	Davies et al. (2020) Australia [35]	Staff of Aboriginal and other community health services who attended ‘We Yarn’ training	*N* = 106 (survey) *N* = 9 (interview) *N* = 48 (observation)	N/A	N/A	Rural NSW community	Gatekeeper
8	Donald et al. (2013) Australia [36]	Mental health workers from community, inpatient, emergency, and school settings	*N* = 242 (basic training) *N* = 55 (enhanced training)	Age range: 20–50 +	N/A	Mental health services	Professional Development
9	Exner-Cortens et al. (2022) Canada [39]	Teachers and school staff	*N* = 26 (Overall sample) *N* = 18 (Control- QPR only) *N* = 8 (Intervention – QPR + NL)	N/A	N/A	Highschool	Gatekeeper Training
10	Gask et al. (2008) Scotland [40]	Trained STORM health workers, allied health managers, and a service user	*N* = 203 (quantitative) *N* = 12 qualitative interviews	M_age_ = 43	Female (73%)	Mental health services	Professional Development
11	Gutierrez et al. (2020) USA [28]	Licensed clinical social workers	*N* = 4	N/A	Female (100%)	Veterans Affair Community-based outpatient clinic (CBOC)	Professional Development
12	Hangartner et al. (2018) USA [5]	Adults working at youth-serving community agencies	*N* = 2,389	M_age_ = 41.42; SD = 13.87	Female (70%)	Youth-serving agencies, schools, and crisis centres in urban community	Gatekeeper
13	Hayes et al. (2008) UK [41]	Prison staff	*N* = 15 (initially trained trainers) *N* = 183 (staff trained by the trainers)	Age range: 22 – 66 years (M_age_ = 39; SD = 5.6 years)	Male (*n* = 117) Female (*n* = 42) *2 people did not specify their gender	Prison	Gatekeeper
14	Hegerl et al. (2019) Germany, Hungary, Portugal, Ireland [42]	Regions in four countries with population size > 150,000, participating in OSPI project	*N* = 4	N/A	N/A	-	Gatekeeper
15	Jones et al. (2018) Australia [37]	Health and human service workers who participated in a suicide prevention training program	*N* = 24	Age Range: 29 – 71 years (M_age_ = 45.5; SD 12.7)	N/A	Regional South Australian community	Professional Development
16	Kalafat & Ryerson (1999) USA [29]	Educators, parents, and students from public high school	*N* = 31	N/A	N/A	Public high school	Gatekeeper
17	La Guardia et al. (2021) USA [24]	Healthcare profession students (undergraduate/graduate students)	*N* = 18	N/A	N/A	Universities (Online)	Professional Development
18	Lindow et al. (2020) USA [11]	High school students who received YAM curriculum at school and received parental consent	*N* = 436	M_age_ = 14.5; SD = 0.65	Female (*n* = 267, 61.3%) LGBT (*n* = 51, 11.8%)	Schools	Curriculum Training
19	Lopes et al. (2012) Australia [38]	Community members	*N* = 10 interviews) *N* = 2 direct observations of workshops	Age range: Early 20s to mid 60s	N/A	Centre for Remote Health (Alice Springs)	Gatekeeper
20	Marshall et al. (2014) USA [10]	Mental health providers who had not completed CAMS	*N* = 139 (total participants) Randomized into: *N* = 69 e-learning *N* = 70 in-person	Age > 50 (N = 59, 42.4%) Age < 50 (N = 80, 57.6%)	Male (*n* = 43, 30.9%) Female (*n* = 96, 69.1%)	Veteran Health Affair Medical Center	Professional Development
21	Matthieu et al. (2008) USA [34]	Veteran Affairs Staff	*N* = 602	M_age_ = 51.2 years	Male (*n* = 373, 63.3%)	Community-based counselling centres (Veteran centres)	Gatekeeper
22	Mishkind et al. (2023) USA [33]	Employees who attended the VitalCog training between October 2018 and January 2021 and responded to pre- and post- evaluation	*N* = 1244	Age range: predominantly between 25–55 (79%)	Female (61%)	Workplace	Gatekeeper
23	Persaud et al. (2019) Guyana [4]	Teachers, administrative staff, and school-allied community workers	*N* = 16	Age range: 18–34 years	Male (31.2%) Female (68.8%)	Private secondary school (rural)	Gatekeeper
24	Pickering et al. (2018) USA [30]	Schools in North Dakota and New York with above-average youth suicide rates	*N* = 20 schools *N* = 5,677 students	N/A	Male (*n* = 2,874, 49.9%) Female (*n* = 2,803, 48.6%)	Schools	Gatekeeper
25	Stewart et al. (2020) USA [31]	Psychologists, psychiatrists, social workers and a licensed mental health clinician	*N* = 12	N/A	N/A	University Counselling Centre	Professional Development
26	Volungis (2020) USA [32]	High school students	Year 1 (*n* = 879) Year 2 (*n* = 755) Year 3 (*n *= 496)	M_age_ (grade 9–12) = 15.5 years Year 1 = Grades 9–12 (13–19 yrs) Year 2 = Grades 10–12 (14–19 yrs) Year 3 = Grades 10–11 (14–18 yrs)	N/A	Schools	Curriculum Training
27	Wexler et al. (2019) USA [23]	Community residents or service providers, aged 15 or older	*N *= 376	3% teens, 13% young adults, 27% adults, 16% elders, and 41% unknown	Female (64%)	Local community villages	Gatekeeper
28	Wexler et al. (2017) USA [22]		*N* = 32	N/A	N/A		

We categorized the suicide prevention training programs into three categories: gatekeeper training, professional development, and school-based curriculum training. Gatekeeper training refers to training programs given to members of the community and community organizations to improve their capacity to identify and respond to individuals at risk of suicide [8]. Members of the community can include general health professionals (e.g., physicians); however, we opt to refer to training for these professionals as professional development because we found some differences in the aims and methods of training programs for this population. More specifically, professional development training is given to current or future professional health/mental health care service providers to improve their clinical competencies in engaging, assessing, and providing care to people who are considered at risk of suicide. Lastly, school-based curriculum training refers to a more universal type of prevention training program that is embedded as part of a school curriculum provided largely to students and/or staff [11].

Gatekeeper training was the most reported type of prevention training (*n* = 12, 43%) in the included papers, summarized in Table 3. Most of these training programs were provided to community members [8, 22, 23, 38]. In those cases, suicide prevention training was often intended to improve awareness and attitudes regarding suicide and skills in referring people at risk of suicide to professional care. Gatekeeper training was also provided to community-based professional service providers who might not have a clinical background, such as crisis center counselors [6, 21], prison staff [41], adults working at youth-serving community agencies [5], workplace employees [33] and veteran affairs staff [34].

**Table 3:** Implementation and evaluation strategy – gatekeeper program

No	Authors	Target	Setting	Implementation					Implementation Evaluation		
				**Training**	**Aim**	**Dose**	**Actor**	**Strategies**	**Outcome**	**Measurement**	**Findings**
1	Cerel et al. (2012) USA [8]	Kentucky community members who received QPR (Question Persuade Refer) training	Community	QPR	Improve knowledge and help seeking behaviour in suicide distressed people	1 day training session from a QPR master trainer or an 8-h self-study course	QPR Master trainer	• Implement train-the-trainer strategy • Use of didactic lecture presentation • Use interactive media • Use of role-play/ behavioral rehearsal	• Feasibility	• Survey (5-point rating scale, pre- and post- evaluation to measure satisfaction)	• Shorter training more feasible • Training worked best for those with no previous suicide prevention gatekeeper training • No differences were noted in outcomes for individuals in which a video or PowerPoint had been used • Role plays associated with lower perceived knowledge, efficacy, and satisfaction
2	Cross et al. (2014) USA [6]	Counselors who had received training for delivering ASIST at 17 Lifeline crisis centers	Crisis centres	ASIST	Improve trainer fidelity (over time), training model adherence	5 days	ASIST Master trainers	• Implement train-the-trainer strategy • Distribute training manual to guide delivery	• Fidelity	• Survey measuring adherence to program (7-item scale) and competency in program delivery (5-item scale) – developed by the program developer	• Previous crisis or suicide prevention training was associated with lower levels of trainer adherence to the ASIST model
3	Cross et al. (2017) USA [21]										• Use of recommended behaviors by counselors was mainly related to trainers' competence in delivery of the program rather than adherence to the program content
4	Davies et al. (2020) Australia [35]	Staff of Aboriginal and other community health services who attended ‘We Yarn’ training	Rural NSW community	We Yarn Training	To provide culturally safe suicide prevention training and improve knowledge and attitude about suicide and suicide prevention for Aboriginal people and those who work with them	1 day (6 h)	Experienced non-Aboriginal suicide prevention trainer and an experienced Aboriginal facilitator	• Develop program content with local stakeholders • Involve local facilitator to deliver training • Involve people with lived experience	• Feasibility	• Survey (pre- and post- evaluation through open-ended questions about pre-workshop expectations, post-workshop suggestions for improvement)	• Consider strategies to engage individuals with low knowledge of suicide prevention but high exposure to individuals experiencing social and emotional challenges • Develop tailored workshops for health professionals that align with a holistic health and cultural framework • Establish prolonged community engagement before and after workshops to better understand the local context and facilitate flexible responses and follow-up activities
5	Hangartner et al. (2018) USA [5]	Adults working at youth-serving community agencies	Youth-serving agencies, schools, and crisis centres in urban community	QPR	Increase suicide prevention awareness and teach adults strategies to identify, persuade, and refer individuals at risk of suicide to helping resources (QPR)	152 2-h, in-person training sessions	QPR-certified instructors	• Use of train-the-trainer strategy • Use of didactic lecture presentation • Use of interactive media • Use of group discussions	• Fidelity	• Random Quality Assurance Checks (10%), with a 20-item dichotomous (yes/no) checklist, independently by two researchers • Survey to assess fidelity of training implementation using a 13-item scale developed by the program developer (assessed post-training)	• Training sessions were implemented with high fidelity to the manualized QPR training protocol (M = 97.4%, SD = 6.3%) • Qualitative data collected suggested that participants wanted additional training opportunities to practice suicide prevention behaviors, such as asking about suicide and liaising with community referral resources
6	Hayes et al. (2008) UK [41]	Prison staff	Prison	STORM	Equip staff with the skills to competently assess and manage suicide risk in prison setting (STORM training)	4 days	Members of research team	• Implement train-the-trainer strategy • Use of didactic lecture presentation • Use of demonstration • Use of role-play/behavioral rehearsal • Use of group feedback	• Acceptability	• Survey (satisfaction with training was measured on a series of scales scored on the same 5-point scale as the ASPS at pre-, post-training, and 6–8 months follow-up)	• The most positive ratings were for the lecture and group feedback portions of the training, followed by the demonstration video and role play • The drop in knowledge and confidence scores between T2 and T3 suggest the importance of refresher training
7	Hegerl et al. (2019) Germany, Hungary, Portugal, Ireland [42]	Regions in four countries with population size > 150,000, participating in OSPI project		OSPI-Europe 4-level suicide prevention program	Improve knowledge, attitude and awareness of suicide and depression of GP, general public, and community facilitators	N/A (differs in each country)	N/A	• Provide educational materials (e.g., emergency cards, posters, and holding public events for the general public) • Implement train-the-trainer strategy • Identify and restrict access to lethal means	• Feasibility • Fidelity	• Interviews, observation, FGD, & progress tracking questionnaires by the process evaluation team. • Checklist for intervention & implementation strategies	• Community Facilitators (CF) training was a successful and sustainable intervention • Different training needs among CF’s with different levels of baseline knowledge must be considered when designing and delivering training • Low fidelity: activities differed for each region as there was no ‘common’ intervention beyond guidance for the use of ‘emergency cards’ distributed via hospitals, and the use of existing helplines
8	Lopes et al. (2012) Australia [38]	Community members	Centre For Remote Health (Alice Springs)	Suicide story training	Provide a culturally sensitive approach to improving skills to work with people at risk, and build a sense of hope	3.5-day workshop	Trained ‘implementers’	• Use of narrative therapy approach • Use of interactive media (e.g., video, music, animation)	• Acceptability	• Documents, direct observation, FGD, and semi-structured interviews used to evaluate key implementation process and content relevance	• Knowledge and understanding increased in trainees due to DVD • Workshop length was sufficient to cover the DVD topics and the pace and flexibility of delivery allowed participants to reflect and open up in their own time without feeling overwhelmed • Recruitment and retention of trainees was largely attributed to cultural consultants and the well-established relationships between trainees and implementers • DVD was not considered a standalone resource • The DVD implementation strategy was accepted well because of its duration and delivery of theory, practical components, and free time, delivered flexibly to cater to diverse trainee groups
9	Matthieu et al. (2008) USA [34]	Veteran Affairs Staff	Community-based counselling centres (Veteran centres)	QPR	Increase awareness of suicide and improve ability to identify and refer veterans at risk for suicide	Community Gatekeeper Training Presentation: 1 h multimedia training Behavioural Rehearsal: five-to-seven minute standardized role play dialogue	Certified QPR Institute, Inc. instructor and doctoral level social worker (MMM)	• Use of didactic lecture presentation • Use of role-play/ behavioral rehearsal	• Acceptability	• Survey (role play scale [21] and Peer Observational Checklist (developed by research team for use in this study) were used)) (assessed at pre- and post-training)	• The clinical staff who participated in the practice session reported that it was acceptable, with proportions ranging from 51.9% to 77.7% whereas the nonclinical cohort rated the behavioral rehearsal practice session higher with proportions ranging from 60.1%–80.3%
10	Mishkind et al. (2023) USA [33]	Employees who attended the VitalCog training between October 2018 and January 2021 and responded to pre- and post- evaluation	Workplace	VitalCog	To provide those in workplace settings with information about suicide and tools to help coworkers who may be at risk for suicide	2 h	Certified Vitalog trainers	• Use of role-play/ behavioral rehearsal • Use of demonstration (i.e., live or video) • Use of participant workbook	• Acceptability	• Survey/ questionnaire was developed by the research team to evaluate perceived general comprehension about suicide prevention, confidence, and comfort level (assessed at pre- and post-training)	The use of synchronous technologies to deliver training virtually ofers organizations and managers fexibility to more efficiently provide training VitalCog offered virtually is as efective as, and potentially has advantages over in-person training
11	Wexler et al. (2019) USA [23]	Community residents or service providers, aged 15 or older	Local community villages	PC CARES	Strengthen local systems of support to: (1) translate scientific prevention research to community members; (2) create shared understanding and collaboration; (3) enable local people to strategically reduce suicide risk factors and increase safety, help-seeking, and support	9- 3-h learning circles delivered over 1 year	Locally trained facilitators	• Develop training content with local stakeholders • Use of group discussion • Implement train-the-trainer strategy • Use of people with lived experience (e.g., personal story-telling)	• Fidelity • Acceptability	• Fidelity tracking sheet using audio transcripts of the learning circles • Surveys were used to measure learning outcomes, suicide prevention behavior, and social impact (assessed at baseline and 3-months after the last learning circle)	• Local facilitators achieved acceptable fidelity to the model (80%) & interpreted the research accurately 81% of the time • Participants appreciated the engaged learning model led by local facilitators, train-the-trainer model worked overall, but participation declined over time • Demonstrated feasibility in small rural communities
12	Wexler et al. (2017) USA [22]								• Acceptability • Fidelity	• Survey (5-point Likert satisfaction scale) assessed at pre- and post-training • Fidelity tracking sheet using audio transcripts	• 90% of facilitators found PC CARES to be a culturally responsive way to engage community members in suicide prevention efforts, and most facilitators (20 of 32) initiated learning circles in their villages within 3 months of the training

Nine papers (32%) reported on professional development training (Table 4). Two papers involved healthcare professional trainees [24, 25]. They were derived from the same study that explored the implementation of an Interprofessional Education (IPE)-informed suicide prevention course in person and online. Most of the professional development programs involved mental health workers (e.g., psychologists, psychiatrists, and clinical social workers) in mental health institutions [36, 40], hospital emergency service departments [27], veteran affairs centers [10, 28], and university counselling centers [31]. One paper reported a prevention training program intended for rural health service workers to improve their clinical skills in engaging with people at risk of suicide in regional South Australia [37].

**Table 4:** Implementation strategy – professional development program

No	Authors	Target	Setting	Implementation Strategy					Implementation Evaluation		
				**Training**	**Aim**	**Dose**	**Actor**	**Strategies**	**Outcome**	**Measurement**	**Findings**
1	Bettis et al. (2020) USA [27]	Psychiatry and psychology trainees, nursing and social work staff	Paediatric behavioural health emergency services	SPI	Improve safety by addressing warning signs, coping skills, identify support persons & psychoeducation relating to restricting access to lethal means	5 × 60 min sessions over 3–5 weeks	University-based trainers	• Identify training needs • Develop training plan • Provide individual/ group feedback	• Appropriateness	• Survey (6-item open-ended survey created by the program developer to explore the benefit & challenges of using SPI)	• SPI provided common language and structure that help to normalize the process of safety planning • SPI is useful for parents in that it “allows for group discussion regarding a plan to go home” and “helps parents to know triggers and what to observe” at home • Long wait times in ES settings add strain on both providers and families, indicating that “after extended waits for evaluation, it can be hard to have patients and families be willing to fully participate in planning.”
2	Cramer et al. (2019) USA [25]	Health profession students	Universities	IPE	Improve suicide prevention core competencies	15 weekly modules	Unspecified course professor	• Conduct pilot investigation to adapt training • Use an interdisciplinary approach • Use of didactic lecture presentation • Use of self-reflection activities	• Adoption • Acceptability	• Survey (assessed through course feedback that explores the usefulness of course training and trainees’ intent to apply course content and resources in future health profession work)	• Overall, students were in agreement with intent to use course content across items (M = 4.04/5.00, SD = 0.65) • Students in the blended course preferred several interactive methods more than students in the online course version (large effects) • Online training scenarios, completion of the Suicide Prevention Resource Center (SPRC) free online strategic planning certificate, and IPE team-based design of a suicide prevention program received the highest ratings for effective learning methods • Free online tools such as FlipGrid and Weebly were rated low relative to other learning methods
3	Donald et al. (2013) Australia [36]	Mental health workers from community, inpatient, emergency, and school settings	Mental health services	Unspecified	Improve knowledge of suicide prevention strategies	Basic training: 1 day Enhanced training: 3 days	Unspecified training team in collaboration with state health department and local MH services	• Develop training program with local stakeholders • Use of interactive media (e.g., video) • Use of role-play/ behavioral exercise	• Penetration	• Survey (exploring the expansion of the organizational network and its purpose in relation to the implementation – assessed pre-training and 3-month follow-up post-training)	• Participants of the organizational development group reported a significantly higher number of organizational links at time 3 (F = 20.39; df = 1; p < 0.001) • Training efforts that embed a process for implementing and maintaining changes in practice following the initial training event have a greater impact than training that does not provide at least some period of support to staff once they return to the field
4	Gask et al. (2008) Scotland [40]	Trained STORM health workers, allied health managers, and a service user	Mental health services	STORM	Develop clinical communication skills	4 days	Pre-existing STORM Consultant Facilitators	• Create supportive organizational structure • Distribute training manual • Encourage local facilitators to tailor training to local needs • Use of role-play/ behavioral rehearsal • Provide individual/group feedback • Use of interactive media • Use of self-reflection activities	• Acceptability • Sustainability • Appropriateness	• Survey (exploring participants' satisfaction with specific aspects of the training through a questionnaire developed by the program developer – assessed pre-training, post-training, and 6-month follow-up) • Interview (exploring how the training has influenced policy, organizational culture, etc. & how appropriate it is for their needs)	• STORM's success was attributed to contextual factors (support from the host organization and favorable policy environment) and intervention-related aspects (adaptability, clinical relevance, and utility) • Networking across organizational structures, as a result of multidisciplinary training sessions, helped to formalise experience and communicate to other practitioners the innovation's usefulness
5	Gutierrez et al. (2020) USA [28]	Licensed clinical social workers	Veterans Affair Community-based outpatient clinic (CBOC)	CAMS-G	Improve clinician adherence in delivering intervention for veterans at risk of suicide	Approx 75 h, (including: CAMS training workshop: 1 day) CAMS-G workshop: 1 day CAMS-G: group ran for 1 year, (only 10 sessions were rated for this study)	The study primary investigator & 2 CAMS master trainer	• Provide supervision of implementation • Use of didactic lecture presentation • Use of demonstration • Use of role-play/ behavioral rehearsal •Provide individual/ group feedback • Use of group discussion	• Fidelity	• Survey (measured through the CAMS Rating Scale (CRS.3) & CAMS-Group Rating Scale) [43, 44]	• Facilitators were not adherent to the treatment manual in the initial session but achieved adherence by the second session and maintained acceptable adherence in all subsequent group therapy sessions • Adherence did not dip when the co-facilitation dyads changed after the sixth group session
6	Jones et al. (2018) Australia [37]	Health and human service workers who participated in a suicide prevention training program	Regional South Australian community	Unspecified	Improve skills and experience for engaging with people at-risk of suicide	1 day (6.5 h)	Multidisciplinary research team	• Use of didactic lecture presentation • Use of reflection activities • Use of interactive media (e.g., scenario video, etc.)	• Adoption • Acceptability	• Interview	• Participants reported using their experience of the training in their roles in the community and see this as supporting the resilience of local communities • This paper highlights the value of coproduction; however, further research is needed to identify whether this translates into changes in practice and reduces the risk or rate of suicide
7	La Guardia et al. (2021) USA [24]	Healthcare profession students (undergraduate/ graduate students)	Universities (Online)	IPE	Enhance knowledge, attitudes, and skills relating to suicide prevention	17 weeks	Unspecified course professor	• Use of pre-recorded lecture • Use of self-reflection activities (e.g., journaling) • Use of interactive media (e.g., Increasing engagement with virtual interactive activities) • Implement activities for participants to create an implementation plan • Provide a completion certificate	• Adoption • Acceptability	• Survey (post course and 3 months follow-up included statements concerning intent to and actual use of suicide prevention skills, & items evaluating the training strategies [25]	• All training techniques, including the interprofessional suicide prevention project, case studies with team virtual report summaries, and the Suicide Prevention Resource Center web-based strategic planning activity, received ratings above the midpoint of the scale, indicating their preference among participants • Quizzes and basic web-based activities, such as the use of FlipGrid.com, were considered the least useful assignments • Multi-year findings highlight the importance of incorporating team-based learning and simulated experiences to enhance self-efficacy in suicide prevention work and develop interprofessional competencies • The in-person course delivery component was perceived as valuable by students • Challenges encountered in delivering Interprofessional Education (IPE) training through online modules included technological failures, disparate access, difficulties in managing power hierarchies, limitations in forming rapport and collaboration without nonverbal cues, and the potential slowing of group processes
8	Marshall et al. (2014) USA [10]	Mental health providers who had not completed CAMS	Veteran Health Affair Medical Center	e-CAMS	Improve clinical competencies to assess, monitor, and intervene patients at risk for suicide	6.5 h – 12 sessions for both the in-person and e-learning options 1 day for in-person e-learning delivered over a three-week period	CAMS Developer	• Use of didactic lecture presentation • Use of demonstrations • Use of interactive media (e.g., video) • Use of case studies • Provide supervision of implementation	• Acceptability • Feasibility	• Survey (feedback form) • FGD	• Short-term and single instance courses, such as e-CAMS, showed more favorable treatment-subgroup interactions than ongoing courses • Improved satisfaction was associated with interactivity, audio, and online discussion, while improved learning outcomes were linked to practice exercises, feedback, and repetition • Implementation barriers included providers' busy schedules and conflicts with training dates, failure to complete the e-learning within the required period and all modules, and lower attendance rates for Coaching Calls (78% were unattended)
9	Stewart et al. (2020) USA [31]	Psychologists, psychiatrists, social workers and a licensed mental health clinician	University Counselling Centre	SPI	Improve safety by addressing warning signs, coping skills, identify support persons & psychoeducation relating to restricting access to lethal means	2 h	Postdoctoral research clinicians supervised by co-authors	• Use of didactic lecture presentation • Use of role play/ behavioral rehearsal • Provide individual/group feedback • Use of group discussions	• Feasibility • Acceptability • Clinical utility (Adoption)	• Survey (Pre- and post-training evaluation survey: (three intervals: pre, immediately after, and follow-up 8 to 10 weeks following training)	• Results suggest that SPI is a feasible, acceptable, and useful suicide intervention tool for a range of counseling center providers with diverse clinical backgrounds to administer & incorporate SPI into routine care • Two-thirds of staff implemented SPI least once. The average perceived utility of the training persisted at the 10-week follow-up assessment, with most providers again reporting that it was useful to them • Future training should emphasize soliciting client feedback about their experience with SPI (e.g., barriers and facilitators of its use)

Seven papers reported on school-based curriculum training (Table 5). All these programs were conducted in secondary school settings [11, 26, 32]. While gatekeeper training programs mostly focused on improving social attitudes and skills to refer at-risk individuals to appropriate care, school-based curriculum training programs typically embedded the training into their curriculum and put greater emphasis on increasing students’ protective factors (i.e., coping, and resilience).

**Table 5:** Implementation strategy – school-based curriculum training

No	Authors	Target	Setting	Implementation					Implementation Evaluation		
				**Training**	**Aim**	**Dose**	**Actor**	**Strategies**	**Outcome**	**Measurement**	**Findings**
1	Bailey et al. (2021) USA [26]	Students attending rural schools	Rural schools	YAM	Improve awareness of risk and protective factors relating to suicide, knowledge of depression and anxiety, enhancing coping and resilience skills	5 × 60 min sessions over 3–5 weeks	University-based trainers (experienced working with youth & trained in YAM and assistants (also non-school based staff)	• Use of didactic lecture presentation • Use of role-play/ behavioral rehearsal • Use of group discussion • Distribute educational material	• Feasibility • Fidelity • Acceptability	• Survey (student ratings to measure acceptability, assessed upon program completion) • Feasibility measured by the proportion of schools willing to participate, fidelity of implementation, student attendance at YAM sessions, and extension agent feedback • Discussions & weekly debrief calls with master trainer	• YAM intervention delivered by trainers and assistants was feasible and acceptable • Weekly debrief assisted with adherence to the protocol • Weekly monitoring visits and quality control questionnaires with extensions and assistants showed no or small difference among sites (adherence) • Students found the training to be acceptable • YAM trainer highlighted 2 challenges to the intervention—logistics and teaching. It is difficult to do both. There were also unexpected expenses to travel to the rural area
2	Exner-Cortens et al. (2022) Canada [39]	Teachers and school staffs	Highschool	QPR and Natural Leader Training (NL)	To improve natural leaders’ ability to support the implementation and sustainment of school-based suicide prevention program	QPR: online 60 min NL: 3 h (2-h asynchronous and 1 h synchronous)	N/A	• Co-create training materials in a multidisciplinary team • Conduct pilot investigation to adapt training • Distribute educational material • Use of roleplay/ behavioral rehearsals • Use of small group discussions • Use of hybrid training	• Feasibility • Acceptability • Utility	• Survey (an anonymous training feedback form post-training) • FGD/interview	• Feedback on the flyer was generally positive, but limited overall • Participants preferred QPR training over the flyer and other types of training they had previously taken • Participants found QPR® appropriate for teachers as it addresses the issue without being traumatizing or overwhelming • Building a team was the most valuable aspect of NL training, increasing participants' comfort to intervene and providing opportunities for role-playing and feedback • Suggestions were made to increase time for implementation planning and role-play, and to include content on strengths in addition to addressing stigma/barriers
3	Kalafat & Ryerson (1999) USA [29]	Educators, parents, and students from public high school	Public High School	ASAP	Ensure that members of the school community possess the necessary skills, structure and support to respond effectively to at risk or suicidal youth	Educators' seminar 2 h; Parents' program 30—minutes to 2 h; Student sessions (duration not specified)	ASAP consultants in collaboration with school	• Distribute educational material • Use of role-play/ behavioral rehearsal • Use of group discussion • Provide supervision during implementation • Provide group/ individual feedback	• Penetration • Adoption/ retention	• Survey (to check whether respondents' school retained the program's component) • Interview (identify variables associated with adoption & retention of the program in the last 8–10 years)	• The majority of respondents reported that their schools had established policies and procedures for addressing at-risk students, suicide attempts, and completed suicides • Structured interviews suggested that a large percentage of schools (89%) were open to adapting the ASAP program to suit their specific needs and context • Indicators of staff commitment included specialized training for all teachers delivering the program, sustained involvement of initially trained teachers, and high enthusiasm for the program • Principals were frequently mentioned as key providers of critical organizational support • The absence of an on-site program advocate to coordinate and maintain the program was cited as the primary reason for discontinuation in the two schools
4	Lindow et al. (2020) USA [11]	High school students who received YAM curriculum at school and received parental consent	Schools	YAM	Raise awareness about risk and protective factors for suicide, and to enhance the skills and emotional resiliency needed to deal with adverse life events, stress, and suicidal behaviors	Five-hourly sessions	Trained YAM facilitators	• Use of interactive media • Use of role-play/ behavioral rehearsal • Distribute educational material	• Feasibility • Fidelity • Acceptability	• Survey (pre-, post-, and 3-month follow-up evaluation, exploring student satisfaction and quality control) • Fidelity form (completed by facilitators at the close of each session [45]	• YAM intervention was feasible to deliver and evaluate in US school settings as determined by school and student recruitment, intervention fidelity, and assessment completion rates • Standardized fidelity assessments provided by YAM facilitators and helpers indicated all but class period duration and missing materials were 90% in accordance with protocol • Post-session assessments and bi- weekly “debriefing” meetings involving study leaders and facilitators likely supported adherence to the protocol
5	Persaud et al. (2019) Guyana [4]	Teachers, administrative staff, and school-allied community workers	Private secondary school (rural)	QPR	To improve knowledge of suicide prevention, positive attitudes towards suicide-related issues, and skills to aid and refer	1 session, 2 h	Certified QPR instructor	• Provide educational material • Use of didactic lecture presentation • Use of group discussion • Use of role-play/behavioral rehearsal	• Fidelity • Acceptability • Feasibility	• Survey checklist (22-items, completed by a researcher observing each training session) • Observation	• Results supports the acceptability and feasibility of the training in this study • Findings support use of cultural adaptations to suicide prevention training programs by integrating stakeholder input during development • Feasibility was further supported by attendance retention rate of 76%, which provides support for the methods designed to retain participant attendance: recruiting participants during a faculty meeting, collaborating with teachers and staff to fit the training into natural gaps in teaching schedules, providing multiple sessions that teachers could sign up for over a week, and providing the training within one session
6	Pickering et al. (2018) USA [30]	Schools in areas in North Dakota and New York with above average youth suicide rates	Schools	Sources of Strength: Peer-led suicide prevention program	Modify peer norms regarding coping and help-seeking and increasing youth-adult connection, particularly among students isolated from adults	Half day training (15 modules)	Program developer (one of the authors)	• Implement the train-the-trainer strategy • Use of messaging activities for dissemination	• Penetration	• Survey (measuring the student network)	• In the first year of a 2-year intervention study, findings show that peer leaders across 20 schools were able to disseminate the Sources of Strength intervention within a few months to substantial portions of their school population • Peer leaders reached students at high risk for suicide (due to past year suicide attempt), with regard to direct peer communication about Sources of Strength and through an interactive activity, similarly to other students
7	Volungis (2020) USA [32]	High school students	Schools	SOS	Increase students’ basic knowledge and self-awareness of depression and suicide	2 days (total of 75 min) through school’s formal advisory program	School counseling team	• Use of didactic presentation • Use of video about peers with lived experience • Use of group discussion • Use of distressing activity • Conduct self-screening activity	• Fidelity • Feasibility	• Survey (pre- and post- evaluation survey/ feedback)	• Fidelity in 1st year was not great, but improved after feedback • The school enhanced depression and suicide awareness throughout the year by incorporating visuals and practices like ACT, encouraging teachers to identify and support distressed students, and involving teachers in program development and feedback • Adjustments made to the psychoeducation program based on feedback from previous pilot studies successfully sustained students' progress in subsequent years

### Quality assessment

Across the 28 papers, a range of study designs were used: qualitative (*n *= 4), randomized-controlled trial (*n* = 2), non-randomized controlled trials (*n* = 3), quantitative descriptive (*n* = 13), and mixed method (*n* = 6). Papers reporting on qualitative studies exhibited relatively good quality when implemented as a single method study [37, 38, 40]. The mixed-method studies sometimes lacked justification for the use of mixed methods and had poor integration of the quantitative and qualitative findings [4, 10, 35]. Overall, the quality of quantitative descriptive studies was lacking. Specifically, there was limited information on population size, making it difficult to determine whether the sample appropriately represented the population [11, 22–24, 28, 33, 39]. Additionally, there was limited information available on the measurement tools, including definitions of the indicators being investigated, and reliability and validity of measurement tools [22, 24, 31, 33–36, 42]. While incomplete data can sometimes be expected, some papers did not provide information on the possible reasons for missing or incomplete data. This issue was also found in randomized and non-randomized controlled trials. The quality assessment of the included can be seen in Supplementary File 1.

### Study findings

#### Implementation strategies

Across the training programs, common strategies included didactic lecture presentation, role-play activities, group discussions, interactive learning methods (e.g., video, animation), and distribution of educational materials. Nonetheless, certain strategies appeared to be more prominently used in particular categories of training programs. Table 6 depicts the range of implementation strategies.

**Table 6:** Implementation strategies by training type

No	Implementation Strategies	Gatekeeper Training		Professional Development Training		School-based Curriculum Training	
		**N**	**References**	**N**	**References**	**N**	**References**
1	Co-create training materials in a multidisciplinary team	0	-	0	-	1	[39]
2	Conduct pilot investigation to adapt training	0	-	1	[25]	1	[39]
3	Conduct self-screening activity	0	-	0	-	1	[32]
4	Create a supportive organizational infrastructure	0	-	2	[36, 40]	0	-
5	Develop training plan	0	-	1	[27]	0	-
6	Develop program content with local stakeholders	1	[35]	1	[36]	0	-
7	Distribute educational material	1	[42]	1	[25]	4	[11, 26, 29, 39]
8	Distribute training manual to guide delivery	2	[6, 21]	2	[27, 40]	0	-
9	Encourage local adaptation of training	0	-	1	[40]	0	-
10	Identify and restrict access to lethal means	1	[42]	0	-	0	-
11	Identify early adopters/local champion	1	[30]	1	[36]	0	-
12	Identify training needs	0	-	1	[27]	0	-
13	Implement activities for participants to create implementation plan	2	[5, 41]	1	[24]	0	-
14	Implement train-the-trainer strategy	7	[5, 6, 8, 21, 30, 41, 42]	0	-	0	-
15	Involve local facilitators to deliver training	4	[22, 23, 30, 35]	0	-	0	-
16	Involve people with lived experience	1	[35]	0	-	1	[29]
17	Provide a skill completion certificate	0	-	1	[24]	0	-
18	Provide group/individual feedback	0	-	1	[27]	1	[29]
19	Provide supervision during implementation	0	-	2	[28, 36]	1	[29]
20	Use an interdisciplinary approach	0	-	1	[25]	0	-
21	Use of case studies material	0	-	3	[10, 37, 40]	0	-
22	Use of demonstration (i.e., live or video)	4	[4, 5, 33, 41]	2	[10, 28]	1	[29]
23	Use of didactic lecture presentation	4	[4, 5, 8, 34]	5	[10, 25, 28, 31, 37]	2	[11, 26]
24	Use of distressing activity	0	-	0	-	1	[32]
25	Use of group discussion	0	-	2	[28, 31]	3	[26, 32, 39]
26	Use of hybrid training	0	-	0	-	1	[39]
27	Use of interactive media (e.g., video, music)	2	[8, 38]	0	-	2	[32, 39]
28	Use of narrative therapy approach	1	[38]	0	-	0	-
29	Use of participant workbook	1	[33]	0	-	0	-
30	Use of pre-recorded lecture	0	-	1	[24]	0	-
31	Use of role-play/behavioral rehearsal	6	[4, 8, 29, 33, 34, 38]	3	[10, 36, 40]	3	[11, 26, 39]
32	Use of self-reflection activities	0	-	4	[24, 25, 37, 40]	0	-

Half of the gatekeeper training programs were implemented via a train-the-trainer strategy. This involved a master trainer training several community members who then provided training to a small group of people within their community [5, 6, 8, 21, 29, 30, 41]. This implementation strategy was less commonly used in professional training and curriculum-based training programs, as trainees from those programs directly applied their knowledge with their patients or students. Next, around 28% (*n* = 4) of the gatekeeper training programs involved collaboration with the community during program delivery, either by involving local facilitators [22, 23, 30, 35] or developing program content with local leaders [35]. Modifying the training program to enhance acceptability and cultural appropriateness was relatively common, observed in 8 out of 12 gatekeeper programs. This involved adjustments to either the content [22, 23, 33, 35, 38, 41, 42] or delivery method [22, 23, 33, 38]. For example, one study implemented the Yarn Training program in Aboriginal community health services and included the Aboriginal ‘Social and Emotional well-being’ health model in their training [35]. Another study in an Indigenous community took a more narrative approach to their training by incorporating film, animation, artwork, music, and interviews [38]. One study implemented a regional-level program that combined various prevention training programs: primary care training for general practitioners, public health awareness program (i.e., providing flyers, posters, and holding public events for the general public, community facilitator training, and intervention for patients, high-risk groups, and their relatives [42].

In gatekeeper training programs, community collaboration is typically included. However, professional development programs primarily consisted of direct and standardized training, where participants received instructions and then applied the skills in their own time [10, 27, 28, 31, 36, 37]. Among the nine professional development training programs examined, implementation strategies mainly involved didactic lecture presentations [10, 25, 28, 31, 37], self-reflection activities [24, 25, 37, 40], and case studies [10, 37, 40]. Notably, there is greater emphasis on establishing supportive organizational infrastructure [36, 40] and providing extended post-training supervision [28, 36] compared to the other two training types. A study conducted by Donald [36] adopted the organization development model, which aimed to enhance training implementation by identifying potential early adopters, fostering deep learning opportunities, strengthening skills through individual projects that facilitate local prevention changes, providing personalised support from expert, and establishing a learning network [34]. In general, the implementation strategies prioritized effective training retention.

When it came to school-based curriculum training, implementation strategies primarily involved the distribution of educational materials [11, 26, 29, 39], use of role play activities [11, 26, 39], and small group discussions [26, 32, 39]. In contrast to professional development programs and gatekeeper training, which typically provided training materials in the form of a training manual, school-based curriculum training sometimes offered flyers or psychoeducation materials. This strategy is distinct, possibly due to the greater emphasis on building coping skills, resilience, and raising awareness about mental health and suicide in school-based training. This was also evident through the inclusion of self-screening activities [32] and videos featuring peers with lived experience [32]. It is worth noting that school-based curriculum training is typically developed by the research team or an organization in collaboration with the educational institution where the program will be implemented. This is because the program will be integrated into the school’s curriculum, which will be delivered by school personnel. For instance, the SOS suicide prevention training program incorporated the school’s formal advisory meeting, which take place multiple times throughout the year [32]. However, it is also possible for the training to be delivered by master trainers, similar to the Youth Aware of Mental Health (YAM) training program reported in two papers [11, 26].

#### Implementation evaluation measurement

Of 28 papers, 21 used a survey as one of their evaluation methods. Thirteen only used a survey as a single method to measure implementation outcome [6, 8, 21, 22, 24, 25, 27, 28, 31, 33, 35, 36, 41], while the remaining eight combined it with other methods of evaluation such as interview [29, 38–40, 42], focus group discussions [10, 26, 38, 39, 42], progress tracking questionnaire [42], observation [4, 38, 42], checklist [42], fidelity tracking sheet [11, 22, 23] and documents [38]. Only three other papers assessed its outcome using a single measure, namely through interview [37], quality assurance checklist [5], and tracking sheet [23]. Although the majority uses survey, there were some slight differences in its reliance to use a single or mixed measures across type of training.

In the case of papers reporting on gatekeeper training and professional development training, over 50% of them utilized a single measurement tool. However, among those that employed mixed methods, gatekeeper training studies tended to incorporate a greater variety of qualitative methods within a single study. For instance, one paper described a controlled community-based intervention study conducted across Germany, Hungary, Portugal, and Ireland, which employed a process evaluation guided by the UK Medical Research Council framework [42]. This evaluation involved the use of progress-tracking questionnaires, observations, and qualitative interviews/focus groups conducted at six-month intervals [42]. These methods aligned with the intended implementation outcomes, primarily focused on assessing acceptability and fidelity. Surveys were commonly employed in both gatekeeper training and professional development training to gain insights into trainees’ satisfaction with specific aspects of the training (e.g., content and delivery) or as a measure of fidelity. School-based curriculum training programs differed slightly from the other two types, as more than half of the studies utilized mixed measurements. Nonetheless, these findings suggest that there is still a heavy reliance on the use of surveys, with only 32% of the included studies reported using qualitative evaluation methods, such as interviews, observation, or focus group discussions.

#### Implementation evaluation outcomes

In terms of implementation evaluation, a broad range of outcomes were assessed. Among the 28 papers, the measured implementation outcomes included: acceptability (*n* = 16), fidelity (*n* = 10), feasibility (*n* = 7), adoption (*n* = 5), penetration (*n* = 3), appropriateness (*n* = 2), and sustainability (*n* = 1). There was some variation in the types of outcomes across the training categories, although acceptability, fidelity, and feasibility were observed at least once across all categories. Tables 3, 3 and 5 presents the implementation outcomes for each training category.

Regarding gatekeeper training programs, most papers reported fidelity [4–6, 21–23, 42] and acceptability [8, 22, 33, 34, 38, 41] as the implementation outcome measures. Acceptability refers to the perception among implementation stakeholders that the provided training is agreeable, satisfactory, or palatable [17]. Fidelity measures the extent to which a training program was implemented as prescribed in the original protocol or as intended by the developer. Out of the 13 papers reporting on gatekeeper training implementation, only two measured more than one implementation evaluation outcome. Hegerl et al.’s study measured fidelity and feasibility through a combination of interviews, observations, focus group discussions (FGDs), and progress tracking [42]. A study conducted by Wexler et al. assessed the implementation evaluation outcomes of PC Cares training based on acceptability and fidelity [23]. It is worth noting that while beyond the scope of this study, most of the papers that assessed a single implementation outcome primarily measured participant-related outcomes, such as knowledge and self-efficacy, which are likely intended to measure the effectiveness of the training program.

The range of measured implementation evaluation outcomes in professional development training programs was more diverse. Similar to gatekeeper training programs, acceptability was the most commonly measured outcome in this type of training [10, 24, 31, 37, 40]. However, adoption was the next frequently assessed [24, 25, 31, 37], indicating a greater focus on determining whether the programs were actually utilized by the trainees. Unlike gatekeeper training, there was a greater emphasis on measuring multiple implementation evaluation outcomes within a single study. Out of the 9 papers, more than 10% of the studies employed more than 2 outcome measures. For instance, Gask et al. evaluated the implementation of STORM training for mental health workers by assessing its acceptability, appropriateness, and sustainability [40]. Although acceptability and appropriateness are conceptually similar, the latter pertains more to the perceived fit, relevance, or compatibility of the training program for the specific population or problem. Two studies measured feasibility, but not as a standalone outcome measure [10, 31]. The remaining four studies that measured only one outcome assessed appropriateness [27], adoption [25], penetration [36], and fidelity [28].

In the case of school-based curriculum training programs, all the studies measured multiple implementation evaluation outcomes. Feasibility [11, 26, 32, 39] and fidelity [4, 11, 26, 32] were equally the most measured outcomes. Additionally, acceptability [11, 26], adoption [29], and penetration [29] were also measured. These outcome measures suggest that the emphasis of these studies is on ensuring that the training program can be practically implemented and adhered to as intended within a school setting. This emphasis may be attributed to the integration of most training programs into the school curriculum and their delivery by school staff.

#### Implementation evaluation contributing factors

The involvement of local facilitators and stakeholders during development and delivery of gatekeeper training programs were highly valued, aiding cultural understanding and participant retention [4, 22, 23, 29, 35, 38]. However, diverse baseline knowledge among trainees and trainers can pose challenges, with prior suicide prevention training experience correlating with lower adherence in trainers [6, 21]. Training programs that allowed for flexible delivery to accommodate different learning pace appeared to increase knowledge and skills in trainees with no prior knowledge [35, 42]. Two papers attributed the decline in knowledge, skill retention [41], and adherence [6] in their study to lack of post-training supervision, highlighting the need for continuous monitoring and refresher courses. Participants favoured shorter duration programs, incorporating lectures, DVD demonstrations, and group feedback, while role-plays were generally appreciated despite one study highlighting its potential negative impact on perceived knowledge [8]. These insights informed the design and implementation of gatekeeper training programs, emphasizing the need for tailored implementation strategies.

The utility of professional development training increased when the program facilitated improved communication between clinician and patients [27, 31]. However, one study on SPI training raised concerns about the time required for implementing the training in clinical setting, which already experience lengthy wait times [27]. Interactive blended training was generally preferred over fully online versions [24, 25]. Trainees emphasized the value of incorporating interprofessional team-based learning [24, 25], case studies [24, 25], practice sessions [10, 24, 25], and activities to ensure implementation post training, such as strategic planning activities [24, 25, 36], to enhance self-efficacy in suicide prevention work and foster interprofessional competencies. Satisfaction with the training was positively associated with interactivity, audio elements, and online discussion, while improved learning outcomes were linked to practice exercises, feedback, and repetition [10]. Another important finding was that strategies promoting organizational support as part of the training contributed to its sustainability and effectiveness [36, 37, 40]. Meanwhile, several implementation barriers that were identified all derived from online training, which includes: providers' busy schedules, incomplete e-learning modules, and low attendance rates of for coaching calls, technical issues, difficulties in managing power hierarchies, limitations in forming rapport and collaboration without nonverbal cue [10, 24].

Lastly, the evaluation of school-based training programs suggests that the establishment of a supportive team and organizational network is important because it enhances participants' comfort in intervening in cases of suicide risk and provides opportunities for role-play and feedback [29, 39]. Moreover, the continued involvement of the initial trainer after the training and the presence of an on-site program advocate through weekly debrief sessions were considered crucial indicators for sustaining the training outcomes/practices [29, 39] and adherence [11, 26]. It is imperative to actively incorporate the feedback from teachers and students in the ongoing development and implementation processes, as it has been shown to enhance training attendance, retention, and overall effectiveness [4, 32, 39]. The training structure should facilitate teachers' and students' attendance. For instance, the study by Persaud and colleagues indicated that recruiting participants during a faculty meeting, collaborating with teachers and staff to fit the training into natural gaps in teaching schedules, offering multiple sessions that teachers could sign up for over a week, and condensing the training into a single session contribute to faculty recruitment and attendance retention [4]. This is especially significant, given that one of the challenges identified by YAM-teacher trainers was the difficulty in managing logistics and teaching responsibilities [26]. Overall, one study summarized the key points highlighted across the implementation evaluation reports by stating that the successful implementation of their training program was attributed to the presence of a local champion, content relevance, and strong institutional support [40].

## Discussion

The purpose of this review was to examine the strategies and evaluation methods that underpinned the implementation of suicide prevention training programs. Given the importance of training programs in translating research into practice, the review contributed to a better understanding of implementation research in this field [46]. This review built on existing literature regarding implementation research, specifically regarding implementation strategies [16] and implementation outcomes [17], by specifying it within the context of suicide prevention training. Our review also provided a more nuanced and detailed approach by distinguishing between training programs based on the target population: gatekeeper training, professional development training, and school-based curriculum training.

### Implementation strategies

A previous Delphi study reported as many as 73 types of general implementation strategies [16]. Among them, 11 strategies appeared in our review, including implementing train-the-trainer strategies, distributing educational material, and identifying early adopters/local champions. However, our review identified additional implementation strategies covering, among others, the use of role-play, case studies, involvement of people with lived experience, and implementation plan development activities – which might be more commonly used in suicide prevention training programs.

There were slight differences in the choice of implementation strategies across training categories. Gatekeeper training typically involved collaboration with community leaders in the early development of the training program and used a train-the-trainer format. This aligned with suggestions from previous studies that highlighted the importance of cultural considerations, particularly in implementing suicide prevention training in rural, indigenous, or minority communities [47–49]. Notably, however, collaboration was also desired by participants from professional development training programs (e.g., interprofessional team-based activity) [24, 25] and school-based curriculum training (e.g., collaboration with teachers) [4, 32, 39]. The critical role of collaboration and team-based learning stemmed from the fact that they provided the chance for trainees to receive feedback [29, 39] while simultaneously creating a network of support which could aid future training sustainability [36, 37, 40]. Thus, this critical mechanism should be included in the development and delivery phases of suicide prevention training programs.

Professional development training programs largely followed a more standardized lecture format, aided with visual and audio case studies and opportunities to implement the training to current practices. A previous study identified that brief video-based suicide prevention training for primary care was a successful method for transferring knowledge, particularly when paired with skills demonstration, lived-experience perspective, and memorable implementation framework [50]. It drew on the benefit of visual concept mapping, an education technique based on constructivist learning theory, on health care training [50, 51]. This mechanism of delivery appeared to yield valuable 'real world' learning outcomes that were appreciated by participants who also appeared to gain significant knowledge as a result.

Most school-based curriculum training programs involved role-play activities for students to re-enact possible scenarios. Interestingly, although role-play activities appeared acceptable for students, there were also mixed responses when these were implemented in training programs with community members or professionals. Participants in one out of three studies that evaluated role-play activities saw it as useful [27], while participants in the other two rated it as the least useful among other training activities [8, 41]. Past studies have highlighted several problems that could arise with role-play, e.g., the extent to which the role-play scenario reflects real-life settings (realism), facilitator competence, and quality of reflection and feedback [52]. Still, further research is needed as only a limited number of studies explicitly evaluated the use of role-play for training purposes.

#### Acceptability, feasibility, and fidelity

Across suicide prevention training programs, acceptability, feasibility, and fidelity appeared to be the most measured implementation evaluation outcomes. Although there are conceptual differences between these three outcomes [17], the included studies often used these terms and the way they were measured interchangeably. For that reason, discussion regarding these outcomes were grouped together.

Adapting the suicide training program to enhance cultural content relevance and delivery strategy has been highlighted as one of the keys to improving implementation outcomes. Cultural adaptation is often reported when programs are implemented for targeted populations from rural [4] or indigenous communities like Aboriginal gatekeepers [35]. However, different population groups across specialties or positions also required distinct training approaches. A national survey of 1000 teachers who participated in a professional development training program identified the importance of forms of training activities, content relevance, and duration of training for teachers [53]. Longer duration training was preferred in that study, but this was not borne out in our review. This difference might be due to content relevance, as the national survey focused on developing the knowledge and skills of classroom teachers in math and science, which are more practical and relevant to their needs. In contrast, suicide prevention training may be less familiar to teachers, potentially resulting in a lower perceived need to apply these skills on a daily basis. Similarly, a study by Cross et al. [6] found that health professionals who previously had attended a crisis or suicide prevention training program tended to show lower adherence and fidelity to a new training program, suggesting that individuals with more knowledge on suicide prevention might have lower perceived need to engage and implement the new training. Thus, it would be wise to consider different strategies needed to address groups with different level of knowledge and training on suicide prevention to improve acceptability and feasibility.

### Adoption and sustainability

The long-term retention and practical application of knowledge and skills acquired through training initiatives were important goals across various disciplines. However, in the field of implementation science research and practice, there had been limited emphasis on evaluating the sustained effectiveness of these acquired knowledge and skills over an extended period [54]. Our review identified a scarcity of studies that measured outcomes pertaining to this, such as adoption and sustainability. Nonetheless, some insights can be drawn from our review.

Involving local champions or opinion leaders in the training process has demonstrated a positive impact on training adoption. This finding aligned with a previous study that highlighted the role of local champions as influential figures capable of promoting and endorsing the training within their respective communities or organizations, thus increasing the likelihood of adoption [55]. Regarding sustainability, post-training supervision and on-going consultation, primarily observed in professional development training programs, appeared to have a positive effect on this outcome. This aligns with the literature highlighting the positive contribution that coaching and mentoring has on long-term applications of various healthcare training [56]. Additionally, our review and the literature [52] indicate the importance of embedding the training program within existing organizational structures so they can enhance its sustainability by integrating it into routine practices.

### Implementation measurement

Similar to findings from a review on knowledge translation [40], our review found a heavy reliance on the use of surveys to measure implementation outcomes, while qualitative evaluation methods, such as interviews or focus groups, were less used. This is unfortunate because qualitative approaches such as interviewing participants are useful for uncovering rich and detailed aspects of the implementation context as well as nuanced participant perspectives on the implementation processes [57]. Most process evaluation strategies were also conducted post-intervention, which might not provide a true process evaluation [40].

Additionally, despite many participants highlighting the need for strategies to improve the adoption and sustainability of the training skills, very few studies assessed it as part of their implementation outcomes. Professional development training programs had more outcomes assessing adoption and it was the only training category that measured sustainability. This finding is consistent with a previous study on implementation research highlighting that the term sustainability appeared more frequently in conceptual papers than actual empirical articles measuring sustainability of innovations/training [17]. Moreover, feasibility and acceptability were often measured through open-ended surveys exploring obstacles in training programs, despite these being two conceptually different outcomes. Thus, future studies of implementation of suicide prevention training programs should clearly define their outcomes and outline strategies to evaluate them.

### Strength and limitation

A major strength of this systematic review is its comprehensive search strategy, which included multiple databases and a citation search, allowing as much relevant studies to be included. The review also presented a clear summary of the types of suicide prevention training implemented, and the evaluation methods employed to assess their implementation. However, a limitation of this review was its inclusion of solely peer-reviewed studies published in English, potentially excluding relevant studies published in other languages or in non-peer-reviewed sources. Despite these limitations, this systematic review provides valuable insights into the implementation and evaluation of suicide prevention training programs and highlights crucial evaluation points that could be utilized to improve future suicide prevention training implementation. For example, the review revealed a scarcity of studies that evaluated the adoption and sustainability of the training outcomes, highlighting the need for future research in this area.

## Conclusions

Our study offers valuable insights into the underlying strategies and evaluation methodologies integral to the successful implementation of suicide prevention training programs. The discerning analysis of distinct training categories reveals discernible patterns wherein certain strategies and evaluative measures manifest with greater frequency and significance. The pivotal role of collaborative engagement among stakeholders, meticulous assessment of content relevance, and consistent post-training supervision emerges as a prevailing thematic triad that transcends categorical boundaries. Furthermore, advocating for a tailored approach to strategy formulation to accommodate varying levels of expertise and proficiency in suicide prevention augments both program acceptability and practicability. Future research should evaluate approaches that facilitate adoption and sustainability of suicide prevention training programs.

## Supplementary Information

Supplementary Material 1

## Data Availability

All data generated or analysed during this study are included in this published article [and its supplementary information files].

## Abbreviations

ASAP: Adolescent suicide awareness program
ASIST: Applied suicide intervention skills training
CAMS-G: Collaborative assessment and management of suicidality – group
e-CAMS: E -collaborative assessment and management of suicidality
IPE: Interprofessional education
MMAT: Mixed methods appraisal tool
PC-CARES: Promoting community conversations about research to end suicide
PGSB: Practice guideline on the assessment and treatment of suicidal behavior
PRISMA: Preferred reporting items for systematic reviews and meta-analyses
PROSPERO: International prospective register of systematic reviews
SFL: Skills for life
SOS: Signs of suicide
SPI: Safety planning intervention
STORM: Skills training on risk management
QPR: Question, persuade, refer
YAM: Youth aware of mental health
