# Supplementary material for: Strategies and evaluation underpinning the implementation of suicide prevention training: a systematic review
Source: BMC Public Health. 2025 Mar 6;25:889. doi: 10.1186/s12889-025-21999-8 (PMC11883942; doi:10.1186/s12889-025-21999-8)
Supplement: Supplementary file 1 — Supplementary Material 1 [file 12889_2025_21999_MOESM1_ESM.docx]

Supplementary Table 1. Mixed-Methods Appraisal Tool Assessment Results

|  | Bailey et al 2021 (26) | Bettis et al 2020  (27) | Cerel et al 2012  (8) | Cramer et al 2019 (25) | Cross et al 2014 (6) | Cross et al 2017 (21) | Davies et al 2020 (35) | Donald et al 2013 (36) |
| --- | --- | --- | --- | --- | --- | --- | --- | --- |
| **Qualitative Studies**   - 1. Is the qualitative approach appropriate to answer the research question?   2. Are the qualitative data collection methods adequate to address the research question?   3. Are the findings adequately derived from the data?   4. Is the interpretation of results sufficiently substantiated by data?   5. Is there coherence between qualitative data sources, collection, analysis and interpretation? |  |  |  |  |  |  | Yes  Yes  Yes  Yes  Yes |  |
| **Randomized Controlled Trials**  2.1. Is randomization appropriately performed?  2.2. Are the groups comparable at baseline?  2.3. Are there complete outcome data?  2.4. Are outcome assessors blinded to the intervention provided?  2.5 Did the participants adhere to the assigned intervention? |  |  |  |  | Yes  Yes  No  Yes  Yes | Yes  Yes  Can’t tell  Can’t tell  Yes |  |  |
| **Non-Randomized Controlled Trials**  3.1. Are the participants representative of the target population?  3.2. Are measurements appropriate regarding both the outcome and intervention (or exposure)?  3.3. Are there complete outcome data?  3.4. Are the confounders accounted for in the design and analysis?  3.5. During the study period, is the intervention administered (or exposure occurred) as intended? |  |  |  | Yes  Yes  No  Can’t tell  Yes |  |  |  | Yes  Can’t tell  No  No  Can’t tell |
| **Quantitative Descriptive**  4.1. Is the sampling strategy relevant to address the research question?  4.2. Is the sample representative of the target population?  4.3. Are the measurements appropriate?  4.4. Is the risk of nonresponse bias low?  4.5. Is the statistical analysis appropriate to answer the research question? | Yes  Yes  Yes  Can’t tell  Yes | Yes  Yes  Yes  Can’t tell  Yes | Yes  Yes  Yes  No  No |  |  |  | Yes  Yes  Can’t tell  Yes  Yes |  |
| **Mixed-Methods**  5.1. Is there an adequate rationale for using a mixed methods design to address the research question?  5.2. Are the different components of the study effectively integrated to answer the research question?  5.3. Are the outputs of the integration of qualitative and quantitative components adequately interpreted?  5.4. Are divergences and inconsistencies between quantitative and qualitative results adequately addressed?  5.5. Do the different components of the study adhere to the quality criteria of each tradition of the methods involved? |  |  |  |  |  |  | No  Yes  No  Can’t tell  Yes |  |
|  | Exner-Cortens et al 2022 (39) | Gask et al 2008  (40) | Gutierrez et al 2020 (28) | Hangart- ner et al 2018 (5) | Hayes et al 2008 (41) | Hegerl et al 2019 (42) | Jones et al 2018 (37) | Kalafat & Ryerson 1999 (29) |
| **Qualitative Studies**   - 1. Is the qualitative approach appropriate to answer the research question?   2. Are the qualitative data collection methods adequate to address the research question?   3. Are the findings adequately derived from the data?   4. Is the interpretation of results sufficiently substantiated by data?   5. Is there coherence between qualitative data sources, collection, analysis and interpretation? | Yes  Yes  No  No  Yes | Yes  Yes  Yes  Yes  Yes |  | Yes  Can’t tell  Yes  No  Yes |  | Yes  Yes  Yes  Yes  Yes | Yes  Yes  Yes  Yes  Yes | Yes  Yes  Can’t tell  Yes  No |
| **Randomized Controlled Trials**  2.1. Is randomization appropriately performed?  2.2. Are the groups comparable at baseline?  2.3. Are there complete outcome data?  2.4. Are outcome assessors blinded to the intervention provided?  2.5 Did the participants adhere to the assigned intervention? |  |  |  |  |  |  |  |  |
| **Non-Randomized Controlled Trials**  3.1. Are the participants representative of the target population?  3.2. Are measurements appropriate regarding both the outcome and intervention (or exposure)?  3.3. Are there complete outcome data?  3.4. Are the confounders accounted for in the design and analysis?  3.5. During the study period, is the intervention administered (or exposure occurred) as intended? |  |  |  |  |  |  |  |  |
| **Quantitative Descriptive**  4.1. Is the sampling strategy relevant to address the research question?  4.2. Is the sample representative of the target population?  4.3. Are the measurements appropriate?  4.4. Is the risk of nonresponse bias low?  4.5. Is the statistical analysis appropriate to answer the research question? | Yes  Can’t tell  No  Can’t tell  No |  | Can’t tell  No  No  Can’t tell  Can’t tell |  | Yes  Yes  Yes  No  Yes | Yes  Yes  Can’t tell  Yes  Yes |  |  |
| **Mixed-Methods**  5.1. Is there an adequate rationale for using a mixed methods design to address the research question?  5.2. Are the different components of the study effectively integrated to answer the research question?  5.3. Are the outputs of the integration of qualitative and quantitative components adequately interpreted?  5.4. Are divergences and inconsistencies between quantitative and qualitative results adequately addressed?  5.5. Do the different components of the study adhere to the quality criteria of each tradition of the methods involved? | Yes  Yes  Yes  Can’t tell  Can’t tell |  |  |  |  | Yes  Yes  Yes  Yes  Yes |  |  |

|  | La Guardia et al 2021 (24) | Lindow et al 2020  (11) | Lopes et al 2012 (38) | Marshall et al 2014 (10) | Matthieu et al 2008 (34) | Mishkind et al 2023 (33) | Persaud et al 2019 (4) | Pickering et al 2018 (30) |
| --- | --- | --- | --- | --- | --- | --- | --- | --- |
| **Qualitative Studies**   - 1. Is the qualitative approach appropriate to answer the research question?   2. Are the qualitative data collection methods adequate to address the research question?   3. Are the findings adequately derived from the data?   4. Is the interpretation of results sufficiently substantiated by data?   5. Is there coherence between qualitative data sources, collection, analysis and interpretation? |  |  | Yes  Yes  Yes  Yes  Yes | Yes  Yes  Can’t tell  No  Can’t tell |  |  | Yes  Yes  No  No  No |  |
| **Randomized Controlled Trials**  2.1. Is randomization appropriately performed?  2.2. Are the groups comparable at baseline?  2.3. Are there complete outcome data?  2.4. Are outcome assessors blinded to the intervention provided?  2.5 Did the participants adhere to the assigned intervention? |  |  |  | Yes  Yes  Can’t tell  Can’t tell  Yes |  |  |  |  |
| **Non-Randomized Controlled Trials**  3.1. Are the participants representative of the target population?  3.2. Are measurements appropriate regarding both the outcome and intervention (or exposure)?  3.3. Are there complete outcome data?  3.4. Are the confounders accounted for in the design and analysis?  3.5. During the study period, is the intervention administered (or exposure occurred) as intended? |  |  |  |  |  |  | Can’t tell  Yes  Can’t tell  No  Yes |  |
| **Quantitative Descriptive**  4.1. Is the sampling strategy relevant to address the research question?  4.2. Is the sample representative of the target population?  4.3. Are the measurements appropriate?  4.4. Is the risk of nonresponse bias low?  4.5. Is the statistical analysis appropriate to answer the research question? | Can’t tell  Can’t tell  Can’t tell  Can’t tell  Yes | Yes  Can’t tell  Yes  Yes  Yes |  |  | Yes  Yes  Can’t tell  Yes  Yes | Yes  Can’t tell  Can’t tell  Yes  No |  | Yes  Yes  Yes  Yes  Yes |
| **Mixed-Methods**  5.1. Is there an adequate rationale for using a mixed methods design to address the research question?  5.2. Are the different components of the study effectively integrated to answer the research question?  5.3. Are the outputs of the integration of qualitative and quantitative components adequately interpreted?  5.4. Are divergences and inconsistencies between quantitative and qualitative results adequately addressed?  5.5. Do the different components of the study adhere to the quality criteria of each tradition of the methods involved? |  |  |  | Can’t tell  No  No  Can’t tell  No |  |  | No  No  No  Can’t tell  No |  |

|  | Stewart et al 2020 (31) | Volungis 2020  (32) | Wexler et al 2017 (22) | Wexler et al 2019 (23) |
| --- | --- | --- | --- | --- |
| **Qualitative Studies**   - 1. Is the qualitative approach appropriate to answer the research question?   2. Are the qualitative data collection methods adequate to address the research question?   3. Are the findings adequately derived from the data?   4. Is the interpretation of results sufficiently substantiated by data?   5. Is there coherence between qualitative data sources, collection, analysis and interpretation? |  |  |  | Yes  Yes  Can’t tell  Yes  Yes |
| **Randomized Controlled Trials**  2.1. Is randomization appropriately performed?  2.2. Are the groups comparable at baseline?  2.3. Are there complete outcome data?  2.4. Are outcome assessors blinded to the intervention provided?  2.5 Did the participants adhere to the assigned intervention? |  |  |  |  |
| **Non-Randomized Controlled Trials**  3.1. Are the participants representative of the target population?  3.2. Are measurements appropriate regarding both the outcome and intervention (or exposure)?  3.3. Are there complete outcome data?  3.4. Are the confounders accounted for in the design and analysis?  3.5. During the study period, is the intervention administered (or exposure occurred) as intended? | Can’t tell  Can’t tell  No  No  Can’t tell |  |  |  |
| **Quantitative Descriptive**  4.1. Is the sampling strategy relevant to address the research question?  4.2. Is the sample representative of the target population?  4.3. Are the measurements appropriate?  4.4. Is the risk of nonresponse bias low?  4.5. Is the statistical analysis appropriate to answer the research question? |  | Yes  Yes  No  Can’t tell  No | Yes  Can’t tell  Can’t tell  No  yes | Yes  Can’t tell  No  No  Yes |
| **Mixed-Methods**  5.1. Is there an adequate rationale for using a mixed methods design to address the research question?  5.2. Are the different components of the study effectively integrated to answer the research question?  5.3. Are the outputs of the integration of qualitative and quantitative components adequately interpreted?  5.4. Are divergences and inconsistencies between quantitative and qualitative results adequately addressed?  5.5. Do the different components of the study adhere to the quality criteria of each tradition of the methods involved? |  |  |  | Yes  Yes  Yes  Yes  No |
